# Primary large B-cell lymphoma of the central nervous system with positive NMDAR antibody: a case report

**DOI:** 10.1186/s12883-022-02821-z

**Published:** 2022-08-12

**Authors:** Xiaoling Li, Mengjiao Sun, Wei Liu, Ning Liu, Boyao Yuan, Xiaolu Su

**Affiliations:** 1 Department of Neurology, Lanzhou University Second Hospital, Cuiyingmen 82, Chengguan District, 730030 Lanzhou, China; 2 Department of Pathology, Lanzhou University Second Hospital, Cuiyingmen 82, Chengguan District, 730030 Lanzhou, China

**Keywords:** Anti-NMDAR, CNS, Lymphoma, Case report

## Abstract

**Background:**

N-methyl-D-aspartate receptor (NMDAR) is an ionotropic glutamate (Glu) receptor that is widely expressed in the central nervous system (CNS), mainly in the hippocampus. We present a case in which the patient had atypical clinical manifestations and was positive for anti-NMDAR antibodies.

**Case presentation:**

A 40-year-old male was admitted to the hospital with “dizziness and double vision for 2 months”. At admission, the patient was lethargic, had short-term memory loss, exhibited loss of orientation (time, place, and person) and calculation ability, and had limited left eye abduction. After admission, serum anti- NMDAR antibody was 1:32, and cerebrospinal fluid was 1:1. Magnetic resonance imaging (MRI) revealed diffuse abnormal signals in the bilateral basal ganglia, thalamus, brainstem, hippocampus, and temporal lobe, with patchy and heterogeneous enhancement. A stereotactic brain biopsy was performed, and the pathological results indicated normal brain tissue. Preliminary diagnosis suggested anti-NMDAR antibody encephalitis. The patient was treated with methylprednisolone combined with intravenous gamma globulin; the symptoms were alleviated, and the patient was discharged. Two months later, the patient’s symptoms worsened, and a second stereotactic brain biopsy was performed. The pathological results showed that the patient had primary diffuse large B-cell lymphoma of the CNS, and the patient was transferred to the Department of Hematology and received chemotherapy combined with rituximab. The patient was in stable condition.

**Conclusions:**

When the primary CNS diffuses large B-cell lymphoma is associated with autoimmune encephalitis, it is very easy to be misdiagnosed. The diagnosis should not be based on the pathological examination that was performed in the early stage of the disease. Therefore, in the diagnosis of immune diseases caused by nervous system infections, it is necessary to dynamically observe the evolution of the disease, perform differential diagnoses when necessary, and ultimately improve our understanding of the disease.

## Background

N-methyl-D-aspartate receptor (NMDAR) is an ionotropic glutamate (Glu) receptor that is widely expressed in the central nervous system (CNS), mainly in the hippocampus. NMDAR is involved in physiological processes such as learning and memory and synaptic plasticity [1]. Primary CNS lymphoma (PCNSL) is a rare type of non-Hodgkin’s lymphoma that affects the brain, spinal cord, and eyes and accounts for approximately 3–5% of intracranial tumors, 1% of all lymphomas, and less than 5% of non-Hodgkin’s lymphoma [2]. The clinical manifestations of PCNSL lack specificity and can be difficult to differentiate from those of diseases such as autoimmune encephalitis combined with cognitive impairment and psychiatric symptoms, thus leading to misdiagnosis; oftentimes, multiple biopsies are required to confirm the diagnosis. Previous studies have reported Hodgkin’s lymphoma combined with anti-NMDAR encephalitis [3], but primary diffuse large B-cell lymphoma (one of the non-Hodgkin lymphomas) of the CNS with anti-NMDAR antibody positivity has not been reported. In this case study, the patient had atypical clinical manifestations and was positive for anti-NMDAR antibodies, leading to the initial misdiagnosis of “anti-NMDAR antibody encephalitis.” The diagnosis was revised after 2 biopsies. The case is summarized herein, to further improve the understanding of PCNSL.

## Case presentation

### General information

The patient, a 40-year-old male, was admitted to the hospital on March 28, 2020, due to dizziness and double vision for 2 months. Two months before admission, the patient had dizziness, headache, and double vision, accompanied by slow responses and somnolence. Magnetic resonance imaging (MRI) in the local hospital revealed diffuse abnormalities in the basal ganglia, thalamus, and brainstem. The patient was diagnosed with viral encephalitis. The patient’s condition worsened after discharge, with significant loss of memory and ability to walk. The patient returned to our hospital for further diagnosis and treatment. The patient had no seizures, fever, or urinary incontinence and normal bowel movements throughout the disease. Since disease onset, the patient had poor spirits, average food intake, and no significant weight loss. The patient was previously in good health, reported no history of hypertension, diabetes, hepatitis, tuberculosis, and food and drug allergies, history of exposure to toxic substances and vaccinations, and family history of similar symptoms. No relevant past interventions were performed before admission.

The results of the physical examination were as follows: body temperature, 36.7 °C; blood pressure, 138/86 mmHg; respiration, 20 beats/min; and pulse, 82 beats/min. An examination of the nervous system indicated that the patient had the following symptoms: confusion of consciousness, occasional ability to respond to questions, lack of cooperation during the physical examination, decreased memory and calculation abilities, poor orientation (time and place), large bilateral pupils (diameter, approximately 3 mm), sensitivity to light, and limited left eye abduction. Extremity muscle strength was grade 4, muscle tension was normal, tendon reflexes of the extremities were normal, and bilateral Barth syndrome and meningeal irritation signs were negative. The patient did not cooperate during the physical examination of sensory and coordinated movement.

### Examinations

**Fig. 1:**
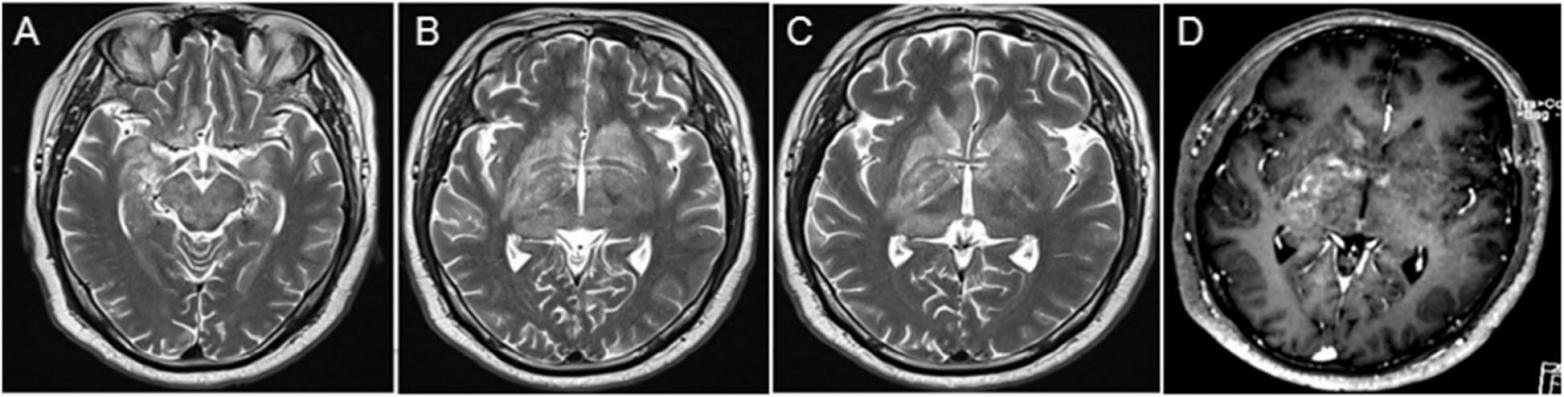
MRI at the first admission. **a-c** T2-weighted sequence, diffuse hyperintense infiltrating lesion, involving the bilateral basal ganglia, thalamus, brainstem, hippocampus, temporal lobe, and left insula. **d** T1 weighted image after contrast: demonstrating heterogeneous enhancement

**Fig. 2:**
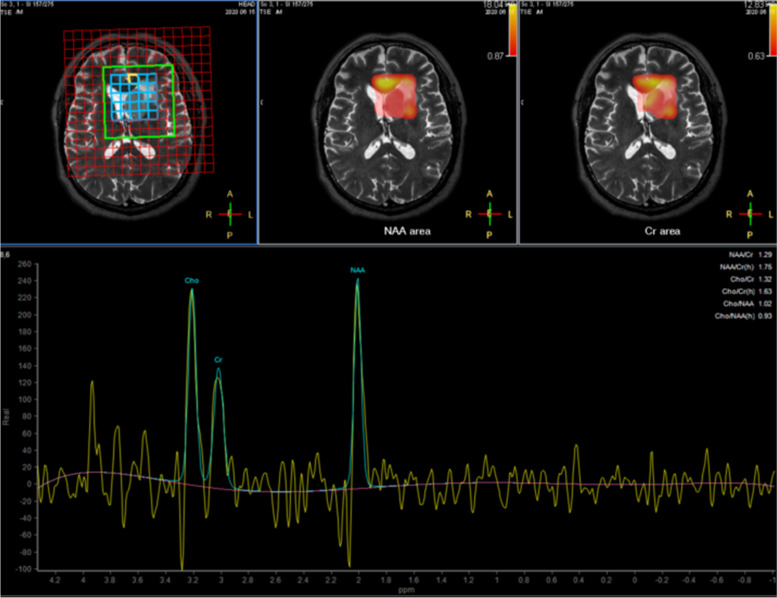
The ^1^H-MRS(TE ms) at the first admission: decreased NAA, increased Cho peak, and towering lipid/lactate peaks can be seen in some areas in the left caudate nucleus. The echo time is 135 ms

After the patient was admitted to our department, MRI revealed diffuse abnormal signals in the bilateral basal nucleus, thalamus, brain stem, hippocampus, temporal lobe, and left insula. Inflammatory lesions were considered, and neoplastic lesions can not be excluded (Fig. 1). The hydrogen proton magnetic resonance spectroscopy showed a decreased N-acetyl -aspartate (NAA) peak, increased choline (Cho) peak, and the presence of lipid/lactate peaks in the left caudate nucleus (Fig. 2). An electroencephalogram (EEG) suggested mild abnormality with predominantly slow waves. Chest computed tomography (CT) indicated no abnormalities. There were no abnormalities in complete blood count, blood biochemistry, erythrocyte sedimentation rate (ESR), rheumatoid factor (RF), C-reactive protein (CRP), anti-nuclear antibody (ANA), anti-cyclic citrullinated peptide (anti-CCP) antibodies anti-streptolysin O (ASO) antibodies, thyroid function, and tumor markers. Based on a lumbar puncture, the cerebrospinal fluid pressure was 160 mmH_2_O, the white blood cell count was 16 × 10^6^/l, the protein concentration was 0.58 g/l, and acid-fast staining was negative. Serum and cerebrospinal fluid autoimmune encephalitis antibody test results (commercial cell-based assay (CBA)) indicated anti-NMDAR antibody positivity (serum anti-NMDAR antibody, 1:32; and cerebrospinal fluid anti-NMDAR antibody, 1:1). A stereotactic brain biopsy was performed, and the pathological results indicated normal brain tissue.

### Diagnosis and differential diagnosis

Based on the pathological results, tumors were excluded, and the diagnostic criteria for anti-NMDR receptor encephalitis, based on the clinical manifestations, imaging examination results, and EEG, were met.

### Treatment

**Fig. 3:**
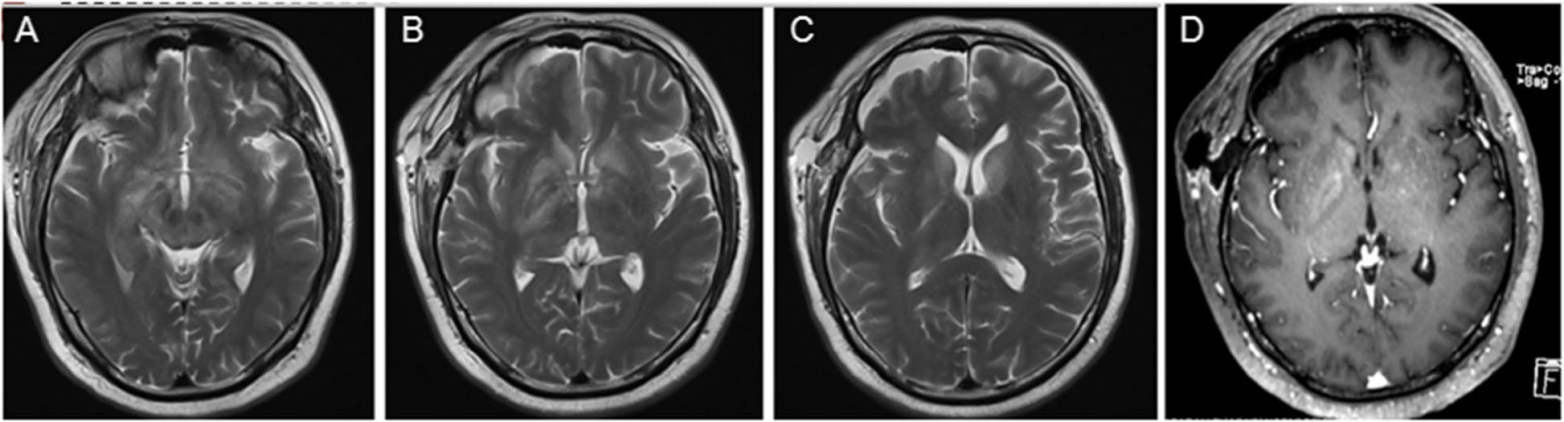
MRI at discharge. **a-c** T2-weighted sequence, the lesions in the bilateral basal ganglia, thalamus, brainstem, hippocampus, and temporal lobe were significantly reduced compared to those in Fig. 1. **d** T1 weighted image after contrast: the enhancement is reduced

The dose of methylprednisolone was gradually reduced after initial treatment with 1000 mg of methylprednisolone and intravenous gamma globulin (0.4 g/kg/d) for 5 days. After 25 days of treatment, the size range of the cranial lesions identified on MRI was smaller than that at admission (Fig. 3), and the patient was recommended for discharge.

### Outcome

**Fig. 4:**
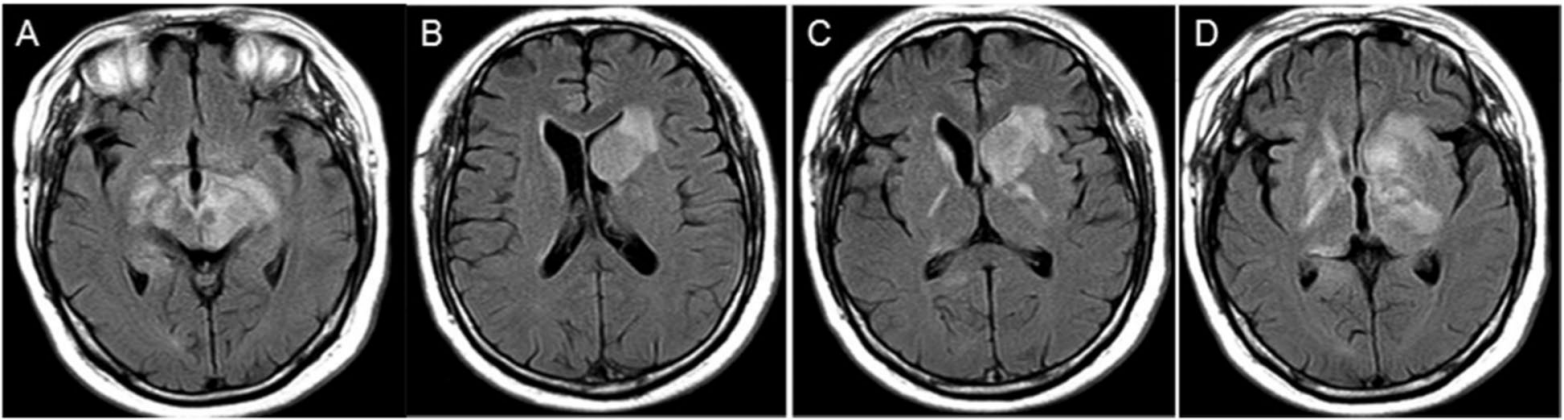
MRI at the second hospital admission. **a-d** FLAIR weighted, the midbrain and basal ganglia demonstrate hyperintensity, with progressive increase

**Fig. 5:**
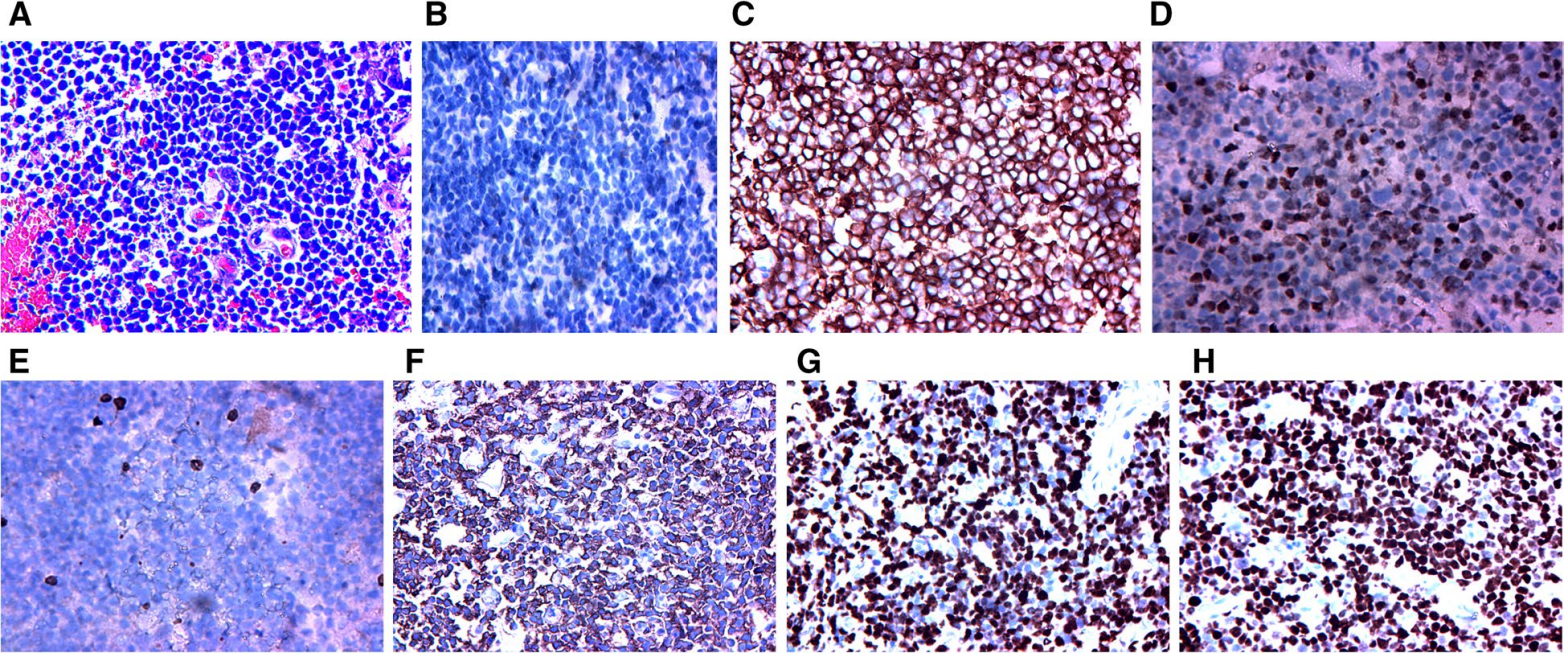
Histopathological stereotactic brain biopsy results. Adjustments of individual color channels were not performed. **a** tumor cells were diffusely distributed, and the abnormally proliferating lymphocytes were primarily B cells. The cells were large and distributed around blood vessels (H&E, 200 ×). **b** no expression of the glial fibrillary acidic protein (GFAP) under microscopy (IHC, 200 ×). **c** strong CD79ɑ expression in Neoplastic B cell membranes (IHC, 200 ×). **d** BCL-6 expression in the nucleus of neoplastic B cells (IHC, 200 ×). **e** scattered CD3-positive interspersed T lymphocytes, suggesting no CD3 expression in neoplastic B cells (IHC, 200 ×). **f** diffuse expression of CD20 in the cell membrane of Neoplastic B cells (IHC, 200 ×). **g** high proliferation index for and high Ki67 expression in tumor cells; and the percent of Ki67-positive cells in the nucleus was 85% (IHC, 200 ×). **h** strong PAX5 expression in the nucleus of neoplastic B cells (H&E, 200 ×)

**Fig. 6:**
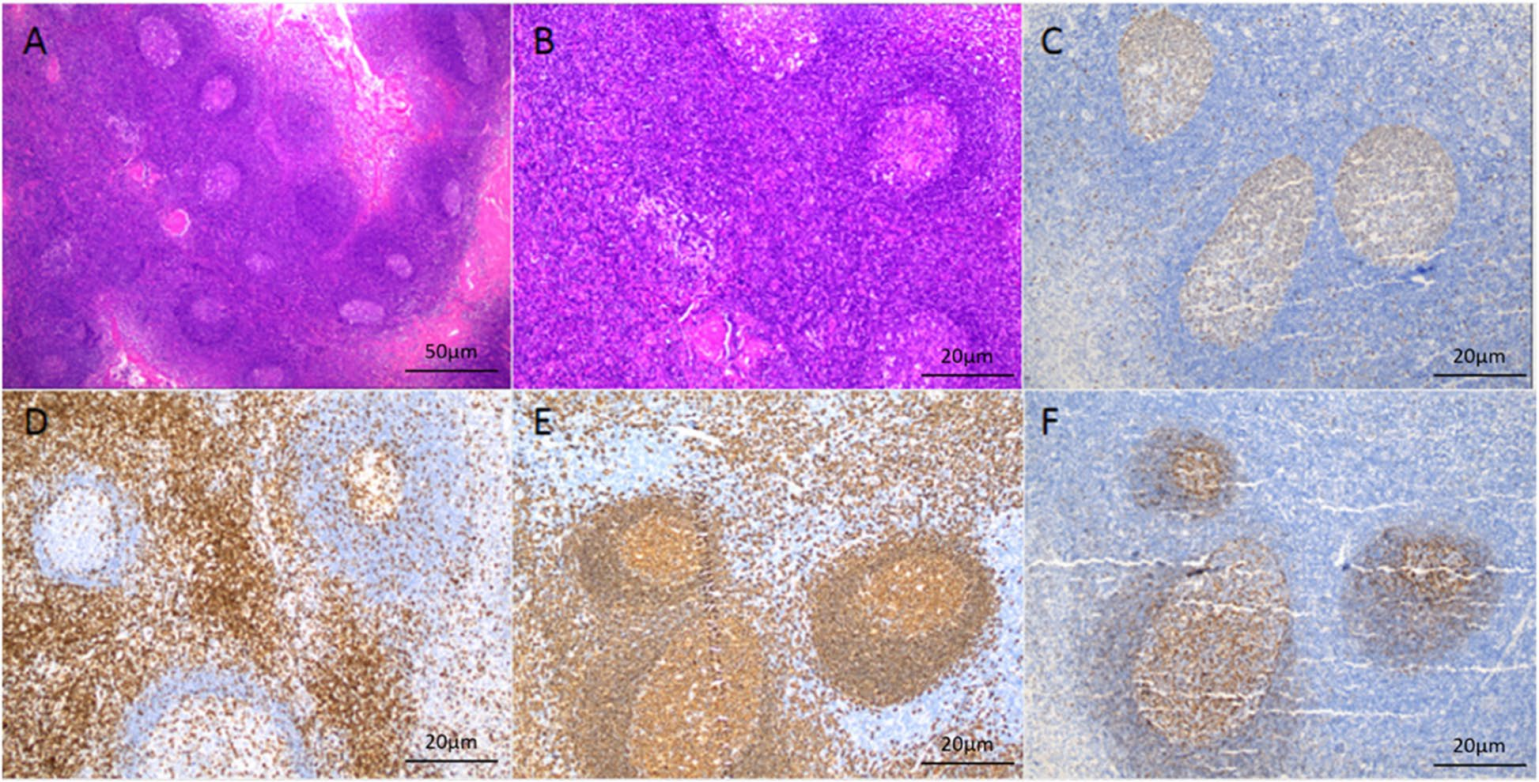
Biopsy results of a right cervical lymph node. Adjustments of individual color channels was performed, there were no threshold manipulation, expansion or contraction of signal ranges and the altering of high signals. **a** lymph node structure exists (H&E, 40 ×). **b** lymph node structure is not destroyed, germinal center, mantle area, cortical area, and paracortical area are visible (H&E, 100 ×). **c** Ki67 showed a normal germinal neutral nucleus proliferation index of 75% (IHC, 100 ×). **d** CD23 staining showed positive T cell membrane/plasma in the paracortical area (IHC, 100 ×). **e** CD20 staining showed positive B cell membranes in the follicular germinal center, mantle area, and cortical area (IHC, 100 ×). **f** CD21 staining showed a positive follicular dendritic network in the germinal center (IHC, 100 ×)

One month after discharge, the patient's condition worsened, and the MRI revealed that the lesion in the head of the left caudate nucleus was significantly enlarged (Fig. 4). A second stereotactic brain biopsy was performed, and the pathological results indicated the proliferation of atypical B lymphocytes in tissues expressing cluster of differentiation 20 (CD20), CD79α, and paired box 5 (PAX5), mainly medium-large cells, accompanied by necrosis and very few T cells; 85% of cells expressed Ki67. The patient was suspected of having primary diffuse large B-cell lymphoma of the CNS (Fig. 5). Positron emission tomography (PET)-CT revealed the following: 1. multiple intracranial high metabolic shadows; and 2. hypermetabolic lymph nodes in the right cervical region. Pathological results of lymph node biopsy suggest reactive hyperplasia in lymph nodes, all of the images were captured by using OLYMPUS (CKX53, PLCN40X,CACHN40XIPC) in a resolution of 4032 × 3024 pixels (Fig. 6). A further request for a bone marrow biopsy was rejected by the patient. The patient was transferred to the Department of Hematology to receive chemotherapy combined with rituximab. The patient was followed up 1 month after discharge. The patient was in stable condition and was followed up regularly. Up to the last follow-up (26 months), the patient was regularly treated with chemotherapy and was in stable condition.

## Discussion and conclusions

Autoimmune encephalitis is an autoimmune disease that has received extensive attention in recent years. Among them, anti-NMDAR encephalitis is the most common and is primarily mediated by NMDAR-targeting immunoglobulin G1 (IgG1) antibodies. There are three different subunits in NMDARs: NR1, NR2, and NR3 [4]. The NMDAR-IgG1 antibodies in cerebrospinal fluid mainly target the NR1 subunit of NMDAR on the postsynaptic membrane of neurons, which are distributed throughout the limbic system and in the basal ganglia and the brainstem. NMDAR-IgG1 antibodies reduce the density of NMDAR on the postsynaptic membrane and disrupt the interaction with ephrin receptor 2 (Ephs2), resulting in the excessive release of glutamate and excitotoxicity and causing characteristic clinical manifestations, i.e., mental and behavioral abnormalities, memory loss, seizures, autonomic dysfunction, and decreased levels of consciousness [5]. Animal experiments have shown that mice with reduced NR1 subunit expression or NR2A subunit deletion exhibit behavioral changes characteristic of schizophrenia [6].

Consistent with the diagnostic criteria for anti-NMDAR encephalitis [7], the clinical manifestations of this patient at admission were diplopia, memory loss, drowsiness, and anti-NMDAR antibody positivity (serum 1:32, cerebrospinal fluid 1:1 (CBA method)); brain tissue was normally based on pathology. Other diseases were excluded. This patient was initially diagnosed with “anti-NMDAR antibody encephalitis.” The patient’s condition improved after treatment with methylprednisolone therapy and intravenous gamma globulin, and a reexamination of the MRI indicated that the lesion area had decreased. After the decrease in methylprednisolone dose at discharge, the patient had recurrent symptoms, and cranial MRI revealed that the lesion in the head of the left caudate nucleus had enlarged significantly (Fig. 4). To confirm the diagnosis, a second stereotactic brain biopsy was performed, and the pathological report indicated PCNSL. The final diagnosis was primary diffuse large B-cell lymphoma of the CNS.

Primary diffuse large B-cell lymphoma of the CNS with anti-NMDAR antibody positivity, as in this case study, has not been reported. Previous studies have reported Hodgkin’s lymphoma and non-Hodgkin’s lymphoma combined with anti-NMDAR antibody encephalitis [8]. However, there is no relevant report on NMDAR antibody-positive PCNSL. PCNSL originates in the CNS. The pathogenesis of PCNSL is currently unclear but may be related to immunodeficiency [9]. The clinical manifestations of PCNSL, such as subacute onset, progressive cognitive impairment, and cranial nerve damage, lack specificity, leading to difficulty in differentiating PCNSL from other diseases, such as autoimmune encephalitis and intracranial primary granuloma. MRI can be normal or may demonstrate hippocampal and insular hyperintensities on T2 and FLAIR weighted images [10]. Fluorodeoxyglucose-positron emission tomography should be considered when MRI is normal. For example, for this patient, the presence of cognitive dysfunction combined with anti-NMDAR antibody positivity (serum, 1:32; cerebrospinal fluid, 1:1) led to an initial misdiagnosis of anti-NMDAR antibody encephalitis. Currently, there is no unified standard for the diagnosis of PCNSL. Although, some features are clues to the diagnosis: CNS lymphomas may present in varied forms, however, some findings can contribute to facilitating the differential diagnosis which includes gliomas, metastases, and inflammatory diseases. The finding of an expansile supratentorial solid lesion hypointense on T2-weighted images, with no signs of necrosis, lipids peak in the solid component, low perfusion, and diffusion restriction, favors the possibility of such diagnosis [11, 12]. In terms of anti-NMDAr encephalitis, most frequently, lesions in grey matter structures located in the limbic system (as the hippocampus) but also in cortical regions (frontal, temporal, parietal, and insular cortices) subcortical nuclei (thalamus, basal ganglia, brainstem) and also in the hemispheric white matter [10, 13]. The diagnosis requires a brain biopsy, and some patients even require multiple biopsies. In this case, the patient was diagnosed after 2 biopsies. The initial treatment with steroids and immunoglobulins may have led to atypical lesions, furthermore, the stereotactic brain biopsy may not precisely puncture the tumor tissue, resulting in normal brain tissue in the first biopsy. The second biopsy was performed two months later to confirm the diagnosis, including the lymph node biopsy. The major limitation of this case is that the follow-up period was relatively short and the intervention in the department of hematology was not recorded in detail. Furthermore, a bone marrow biopsy was not performed.

The following aspects of the case reported herein are worth discussing. (1) There are various imaging manifestations of PCNSL, and MRI manifestations: T1-weighted sequences are iso- or slightly hypointense, most T2-weighted sequences are iso- or slightly hyperintense, FLAIR sequences are slightly hyperintense, and DWI is iso- or slightly hyperintensity. The corresponding area of the ADC presents a low signal. After injection of contrast agent, either nodular and mass-like enhancement, or heterogeneous enhancement can be observed [14]. The MRI presented multiple lesions, with low T1 signal and high T2 signal in bilateral basal ganglia, thalamus, brainstem, and other parts, slightly high signal on FLAIR, and heterogeneous enhancement. When early PCNSL enhancement is atypical, differentiation from other immune-mediated diseases, such as limbic encephalitis, vasculitis, and systemic lupus erythematosus, is necessary. PCNSL is common in patients without immunodeficiency situations, like immunodeficiency virus (HIV), post-organ transplantation, and Epstein-Barr virus (EBV) [15]. Upregulated antigens and/or stimuli from the EBV gene may induce high-level somatic mutations in lymphoid tissues such as lymphocytes or germinal centers, and B cells from mutated lymphoid tissue can transform into lymphoma cells [16]. Victor et al. reported a case of herpes simplex encephalitis complicated with PCNSL in a distal right temporal lobe lesion [17]. It is speculated that the viral infection of neurons may lead to lymphocyte proliferation and mutation or produce local immune system changes, leading to the occurrence of large B-cell lymphoma. In a prospective study, autoantibodies against NMDA receptors were found in the cerebrospinal fluid of 60% of patients with recurrent herpes simplex virus (HSV) encephalitis [18]. Therefore, in this patient, the early immature B-cell-mediated antigen–antibody response may have generated an immune response against the NMDA receptors distributed on the surface of neurons in the temporal lobe, resulting in the production of NMDA receptor antibodies. (2) The importance of cerebrospinal fluid analysis was not fully recognized in the diagnosis of this patient. In the cerebrospinal fluid of this patient, there were 16 × 10^6^ white blood cells per liter, and a cytological examination was not performed. Fifteen percent of PCNSL patients can be diagnosed by detecting immature lymphocytes, with heterogeneous and pachychromatic nuclei, using cerebrospinal fluid cytology, and a biopsy can be avoided [19]. Flow cytometry immunophenotyping and immunoglobulin gene family analysis of cerebrospinal fluid can distinguish malignant and reactive B lymphocytes. Studies have found that potential PCNSL biomarkers include microRNAs (miR-21, miR-101, and 548b). Among them, miR-222 has a certain value for the early detection of diffuse large B-cell lymphoma [20], as do soluble CD19, interleukin (IL-10), and chemokine ligand 13 (CXCL13), but further validation and screening are required to replace brain biopsy [21, 22]. (3) In this case, the use of steroids and gamma globulin after the initial diagnosis may have caused atypical pathological changes that led to 2 biopsies for diagnosis [4]. This indicates that repeated biopsies may be required to confirm a diagnosis of PCNSL.

Finally, the initial misdiagnosis of this patient indicates that when using autoantibody positivity as part of the clinical diagnostic criteria, clinical manifestations, imaging characteristics, routine cerebrospinal fluid tests, and cytology should also be considered for a comprehensive diagnosis. A diagnosis based only on anti-NMDAR antibody positivity lacks comprehensive differentiation. In addition, the initial use of large doses of steroids and gamma globulin reduced the size range of lesions on cranial MRI, having a certain therapeutic effect, but masked the true condition, leading to repeated biopsies to confirm the diagnosis.

In conclusion, we report a case in which the patient had atypical clinical manifestations and was positive for anti-NMDAR antibodies. When the primary CNS diffuses large B-cell lymphoma associated with autoimmune encephalitis, it is very easy to be misdiagnosed. Therefore, in the diagnosis of immune diseases caused by nervous system infections, it is necessary to dynamically observe the evolution of the disease, perform differential diagnoses when necessary, and ultimately improve our understanding of the disease.

## Data Availability

All data generated or analyzed during this study are included in this published article.

## Abbreviations

NMDAR: N-methyl-D-aspartate receptor;
CNS: Central nervous system;
PCNSL: Primary central nervous system lymphoma;
MRI: Magnetic resonance imaging;
EEG: Electroencephalogram;
CT: Computed tomography;
ESR: Erythrocyte sedimentation rate;
RF: Rheumatoid factor;
CRP: C-reactive protein;
ANA: Anti-nuclear antibody;
CCP: Cyclic citrullinated peptide;
ASO: Anti-streptolysin O;
CBA: Commercial cell-based assay;
PAX5: Paired box 5;
CD20: Cluster of differentiation 20;
IgG1: Immunoglobulin G1;
HIV: Human immunodeficiency virus;
EBV: Epstein-Barr virus;
HSV: Herpes simplex virus;
IL: Interleukin;
CXCL13: Chemokine ligand 13

## References

[1] Hansen KB, Yi F, Perszyk RE, Furukawa H, Wollmuth LP, Gibb AJ (2018). Structure, function, and allosteric modulation of NMDA receptors. J Gen Physiol.

[2] Cheng G, Zhang J (2019). Imaging features (CT, MRI, MRS, and PET/CT) of primary central nervous system lymphoma in immunocompetent patients. Neurol Sci.

[3] Zandi MS, Irani SR, Follows G, Moody AM, Molyneux P, Vincent A (2009). Limbic encephalitis associated with antibodies to the NMDA receptor in Hodgkin lymphoma. Neurology.

[4] Alexopoulos H, Dalakas MC (2019). The immunobiology of autoimmune encephalitides. J Autoimmun.

[5] Zandi MS, Paterson RW, Ellul MA, Jacobson L, Al-Diwani A, Jones JL (2015). Clinical relevance of serum antibodies to extracellular N-methyl-D-aspartate receptor epitopes. J Neurol Neurosurg Psychiatry.

[6] Miyamoto Y, Yamada K, Noda Y, Mori H, Mishina M, Nabeshima T (2001). Hyperfunction of dopaminergic and serotonergic neuronal systems in mice lacking the NMDA receptor epsilon1 subunit. J Neurosci.

[7] Dalmau J, Graus F (2018). Antibody-mediated encephalitis. N Engl J Med.

[8] Guo J, Wang Z, Zhang S, Wang D, Tian L, Zhu H (2020). A case report of non-Hodgkin's lymphoma complicated with anti-N-methyl-D-aspartate receptor encephalitis. J Stroke Neurol Disord.

[9] Korfel A, Schlegel U, Johnson DR, Kaufmann TJ, Giannini C, Hirose T (2017). Case-based review: primary central nervous system lymphoma. Neurooncol Pract.

[10] Greiner H, Leach JL, Lee KH, Krueger DA (2011). Anti-NMDA receptor encephalitis presenting with imaging findings and clinical features mimicking Rasmussen syndrome. Seizure.

[11] Reis F, Schwingel R, Nascimento F (2013). Central nervous system lymphoma: iconographic essay. Radiologia Brasileira..

[12] Schwingel R, Reis F, Zanardi VA, Queiroz LS, França MC (2012). Central nervous system lymphoma: magnetic resonance imaging features at presentation. Arq Neuropsiquiatr.

[13] Haberlandt E, Bast T, Ebner A, Holthausen H, Kluger G, Kravljanac R (2011). Limbic encephalitis in children and adolescents. Arch Dis Child.

[14] Barajas RF, Politi LS, Anzalone N, Schöder H, Fox CP, Boxerman JL (2021). Consensus recommendations for MRI and PET imaging of primary central nervous system lymphoma: guideline statement from the International Primary CNS Lymphoma Collaborative Group (IPCG). Neuro Oncol.

[15] Franca RA, Travaglino A, Varricchio S, Russo D, Picardi M, Pane F (2020). HIV prevalence in primary central nervous system lymphoma: a systematic review and meta-analysis. Pathol Res Pract.

[16] Gandhi MK, Hoang T, Law SC, Brosda S, O'rourke K, Tobin JWD (2021). EBV-associated primary CNS lymphoma occurring after immunosuppression is a distinct immunobiological entity. Blood.

[17] Li V, Levine AB, Gooderham PA, Yip S, Chew J (2018). Case of primary central nervous system lymphoma arising at site of remote herpes encephalitis. World Neurosurg.

[18] Armangue T, Leypoldt F, Málaga I, Raspall-Chaure M, Marti I, Nichter C (2014). Herpes simplex virus encephalitis is a trigger of brain autoimmunity. Ann Neurol.

[19] Citterio G, Calimeri T, Ferreri AJM (2018). Challenges and prospects in the diagnosis and treatment of primary central nervous system lymphoma. Expert Rev Neurother.

[20] Thapa DR, Hussain SK, Tran WC, Dʼsouza G, Bream JH, Achenback CJ (2014). Serum microRNAs in HIV-infected individuals as pre-diagnosis biomarkers for AIDS-NHL. J Acquir Immune Defic Syndr.

[21] Fischer L, Korfel A, Pfeiffer S, Kiewe P, Volk HD, Cakiroglu H (2009). CXCL13 and CXCL12 in central nervous system lymphoma patients. Clin Cancer Res.

[22] Shao J, Chen K, Li Q, Ma J, Ma Y, Lin Z (2020). High level of IL-10 in cerebrospinal fluid is specific for diagnosis of primary central nervous system lymphoma. Cancer Manag Res.

